# The Freiburg Index of Post‐TIPS Survival accurately predicts mortality in patients with acute decompensation of cirrhosis

**DOI:** 10.1111/liv.16098

**Published:** 2024-09-09

**Authors:** Eric Kalo, Lukas Sturm, Michael Schultheiss, Oliver Moore, Rajiv Kurup, Chiara Gahm, Scott Read, Marlene Reincke, Jan Patrick Huber, Lukas Müller, Roman Kloeckner, Jacob George, Robert Thimme, Dominik Bettinger, Golo Ahlenstiel

**Affiliations:** ^1^ Blacktown Mount Druitt Clinical School and Research Centre Western Sydney University Blacktown New South Wales Australia; ^2^ Blacktown Hospital Western Sydney Local Health District Blacktown New South Wales Australia; ^3^ Department of Medicine II, Medical Center University of Freiburg, Faculty of Medicine University of Freiburg Freiburg Germany; ^4^ Berta‐Ottenstein‐Programme, Faculty of Medicine University of Freiburg Freiburg Germany; ^5^ Storr Liver Unit Westmead Institute for Medical Research Westmead New South Wales Australia; ^6^ Department of Diagnostic and Interventional Radiology Medical Center University of Mainz, Faculty of Medicine, University of Mainz Mainz Germany; ^7^ Institute of Interventional Radiology University Hospital Schleswig‐Holstein Lübeck Germany; ^8^ Department of Gastroenterology & Hepatology The University of Sydney, Westmead Hospital Westmead New South Wales Australia

**Keywords:** acute‐on‐chronic liver failure, survival, transjugular intrahepatic portosystemic shunt

## Abstract

**Introduction:**

The recently developed Freiburg Index of Post‐TIPS Survival (FIPS) allows improved risk classification of patients with decompensated cirrhosis allocated to transjugular intrahepatic portosystemic shunt (TIPS) implantation. This study investigated the prognostic value of the FIPS in patients hospitalized with acute decompensation of cirrhosis (AD), outside the setting of TIPS implantation.

**Methods:**

A total of 1133 patients with AD were included in a retrospective, multi‐centre study. Ninety‐day, 180‐day and 1‐year mortality were recorded and the FIPS' performance in predicting mortality at these time points was analysed using ROC analyses.

**Results:**

Ninety‐day, 180‐day and 1‐year mortality were 17.7%, 24.4% and 30.8%. Uni‐ and multivariable Cox regression models showed that the FIPS independently predicted 1‐year mortality in the study cohort (HR 1.806, 95% CI 1.632–1.998, *p* < .0001). In ROC analyses, the FIPS offered consistently high performance in the prediction of mortality within 1 year after AD (area under the receiver operator characteristic [AUROC]: 1‐year mortality .712 [.679–.746], 180‐day mortality .740 [.705–.775] and 90‐day mortality .761 [.721–.801]). In fact, in the subgroup of patients presenting with variceal bleeding, the FIPS even showed significantly improved discriminatory performance in the prediction of long‐term mortality (AUROC 1‐year mortality: .782 [.724–.839]) in comparison with established prognostic scores, such as the CLIF‐C AD score (.724 [.660–.788], *p* = .0071) or MELD 3.0 (.726 [.662–.790], *p* = .0042).

**Conclusions:**

The FIPS accurately predicts mortality in patients with AD and seems to offer superior prognostication of long‐term mortality in patients with variceal bleeding.

**Abbreviations**
ACLF: acute‐on‐chronic liver failure
AD: acute decompensation (of cirrhosis)
AUROC: area under the receiver operator characteristic
CLIF‐C AD score: Chronic Liver Failure‐Consortium Acute Decompensation score
EASL: European Association for the Study of the Liver
FIPS: Freiburg Index of Post‐TIPS Survival
HE: hepatic encephalopathy
MASLD: metabolic dysfunction associated steatotic liver disease
MELD: Model for End‐Stage Liver Disease
SBP: spontaneous bacterial peritonitis
TIPS: transjugular intrahepatic portosystemic shunt

**Lay Summary**

Patients with advanced liver disease often experience sudden worsening of their condition. A new tool called the Freiburg Index of Post‐TIPS Survival (FIPS) can help identify which hospitalized patients, due to worsening of their liver disease, are at high risk of death. The FIPS proved to be particularly accurate for predicting long‐term outcomes, especially for patients with variceal bleeding.

## 1 INTRODUCTION

The occurrence of acute decompensation (AD) is an important landmark in the disease course of cirrhosis, as it is associated with significant morbidity and mortality.^1^ Patients with AD who present with acute‐on‐chronic liver failure (ACLF), a distinct syndrome of multi‐organ failure, exhibit a very poor prognosis and high short‐term mortality. In contrast, the outcome of patients with AD who do not fulfil the criteria of ACLF varies significantly.^2^ Hence, precise risk‐stratification of these patients is an important element in the clinical management of AD. Several prognostic scores have been utilized in this context. For many years, the Child–Pugh score has prevailed in the prognostic grading of patients with cirrhosis.^3^ Further, the Model for End‐Stage Liver Disease (MELD) as well as the adjusted MELD‐sodium and MELD 3.0, used for the prediction of short‐term mortality in patients on the liver transplant waitlist, are widely utilized for prognostic assessment of patients with decompensated cirrhosis outside the transplant setting.^4^, ^5^, ^6^ In 2015, the Chronic Liver Failure‐Consortium Acute Decompensation (CLIF‐C AD) score, a bespoke prognostic model for improved risk assessment of patients with AD, was published.^7^ Whilst all of these scores have their own advantages and drawbacks, survival prognostication of patients with AD, especially in the long term, remains a challenge, which is due to the considerable clinical dynamics inherent to this collective of patients.^8^ Hence, there is an ongoing need to evaluate novel scoring systems in order to further optimize risk‐stratification of patients with AD. Recently, the Freiburg Index of Post‐TIPS Survival (FIPS) was established that allows significantly improved prognostic assessment in patients with cirrhosis allocated to transjugular intrahepatic portosystemic shunt (TIPS) implantation due to refractory ascites or for secondary prophylaxis of variceal bleeding.^9^ So far, the utility of the FIPS beyond TIPS placement is unclear, as it has not been thoroughly investigated in other clinical settings.^10^ Therefore, the aim of this study was to investigate the FIPS' prognostic value in patients with AD.

## 2 METHODS

### 2.1 Patient selection and data collection

**FIGURE 1:**
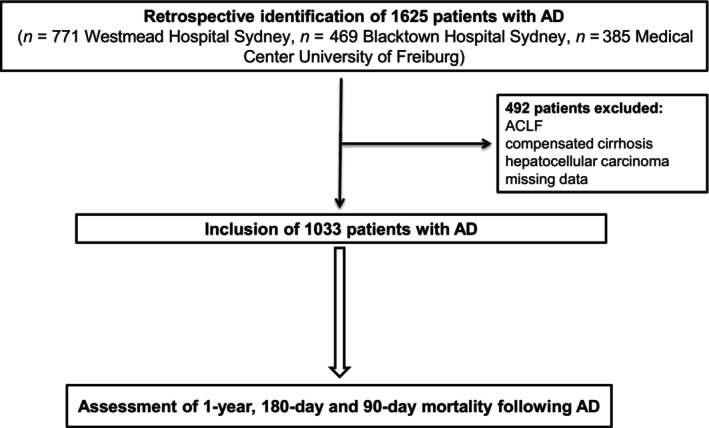
Patient selection. A total of 1625 patients hospitalized due to AD (ascites, SBP, HE, variceal bleeding) at three tertiary care centers were retrospectively identified. 492 patients lacking these manifestations of AD, with ACLF, with hepatocellular carcinoma or with missing medical data were excluded. Eventually, a total of 1133 patients with AD were included in the study. ACLF, acute‐on‐chronic liver failure; AD, acute decompensation.

Patient selection is summarized in Figure 1. A total of 1625 patients hospitalized due to complications of cirrhosis at three tertiary care centers (Westmead Hospital [*n* = 771] and Blacktown Hospital [*n* = 469] of the Western Sydney Local Health District, Australia, and Medical Center University of Freiburg, Germany, [*n* = 385]) between January 2010 and December 2020 were retrospectively identified in the medical records. The following manifestations of AD at the time of hospital admission were assessed, following the definitions of the European Association for the study of the Liver (EASL)^11^: ascites grade 2 or 3, variceal bleeding, overt hepatic encephalopathy (HE) according to the West Haven criteria and spontaneous bacterial peritonitis (SBP). Next, 492 patients lacking these manifestations of AD, fulfilling the criteria for ACLF as defined by the EASL,^2^ with hepatocellular carcinoma or with missing parameters for prognostic score calculation were excluded. Eventually, a total of 1133 patients with AD were included in the study.

The study was an observational analysis. The included patients' clinical data and survival following study inclusion were reviewed in the medical records and with the help of civil registries. Primary endpoint was 1‐year mortality, secondary endpoints were 180‐day mortality and 90‐day mortality. In addition, liver transplantation and TIPS implantation within 1 year after study inclusion were recorded.

### 2.2 Ethics statement

The study was approved by the Human Research Ethics Committee of Western Sydney Local Health District (HREC 2021/ETH00149) and the Ethics Committee of the University of Freiburg (no. EK 21‐1074) and is in accordance with the Declaration of Helsinki. Due to the retrospective design of the study, informed patient consent was waived. The study was conducted following the STROBE guidelines.^12^

### 2.3 Statistical analyses

Continuous variables were expressed as median with interquartile range, categorial variables as frequency and percentage unless stated otherwise. Group differences were determined using chi‐squared or Wilcoxon rank sum tests as appropriate, as there was no Gaussian distribution of the data, which was confirmed by the Shapiro–Wilk test. FIPS, MELD, MELD‐sodium, MELD 3.0, Child–Pugh score and CLIF‐C AD score at the time of study inclusion were calculated for each patient according to the formulas presented in the given references.^3^, ^4^, ^5^, ^6^, ^9^, ^13^ Laboratory parameters for prognostic score calculation were collected within the first 24 h from admission. Survival during the 1‐year observation period was explored using the Kaplan–Meier method and Log‐rank tests. Uni‐ and multivariable Cox regression models (forward variable selection, *p*(in) < .05, *p*(out) > .10, likelihood ratio) were applied to identify prognostic factors. The investigated prognostic scores' discriminatory performance in the prediction of mortality was measured by receiver operating characteristic (ROC) analyses. The areas under the ROC (AUROC) of Child–Pugh score, MELD scores and CLIF‐C AD score were compared against the FIPS score using the nonparametric method by DeLong et al.^14^ Analogous to the original publication of the FIPS, the 85th percentile of the FIPS in the study cohort was evaluated as cut‐off value for risk‐stratification of patients with AD (the rationale for using the 85th percentile is discussed in the original publication of the FIPS).^9^ A *p*‐value <.05 was considered statistically significant. Statistical analyses were performed with STATA® (Version 17.0, Stata Corp LLC., Texas, USA), SPSS® (Version 28.0, IBM, New York, USA) and GraphPad Prism® (Version 9.3, GraphPad Software, California, USA).

## 3 RESULTS

### 3.1 Baseline characteristics and follow‐up data

Baseline characteristics and follow‐up data of the included patients are summarized in Table 1. The median age of patients was 59 (51–67) years, 70.0% of patients was male. Alcohol‐related liver disease was the leading aetiology of cirrhosis with 44.7% of patients, whilst viral liver disease and metabolic dysfunction associated steatotic liver disease (MASLD) accounted for 29.5% and 11.7% of patients, respectively. Ascites was the most common manifestation of AD (71.5% of patients), followed by variceal bleeding (32.1%), HE (30.8%) and SBP (12.1%). The patients had a median FIPS of −.27 (−1.15–.51), a MELD score of 13 (10–19) and a CLIF‐C AD score of 52 (46–59). 52.6% of patients were staged Child‐Pugh class B, followed by Child–Pugh class C (36.3%) and Child–Pugh class A (11.1%).

**TABLE 1:** Baseline characteristics and follow‐up data in the study cohort.

Parameter	Patients (*n* = 1133)
Age [years]	59 (51–67)
Gender	
Female	340 (30.0)
Male	793 (70.0)
Center	
Westmead	442 (39.0)
Blacktown	403 (35.6)
Freiburg	288 (25.4)
Aetiology of liver disease	
Alcohol‐related	507 (44.7)
HBV	67 (5.9)
HCV^a^	267 (23.6)
MASLD	132 (11.7)
Other/cryptogenic	160 (14.1)
Signs of AD at inclusion	
Ascites	810 (71.5)
grade 2	331 (40.9)
grade 3	476 (58.8)
Variceal bleeding	364 (32.1)
HE	349 (30.8)
SBP	137 (12.1)
Number of signs of AD^c^	
Single	712 (62.8)
Two or more	421 (37.2)
Laboratory parameters	
Haemoglobin [mg/dl]	10.8 (8.8–12.6)
Platelets [10^3^/μl]	110 (72–164)
WBC [10^3^/μl]	7.1 (4.8–10.7)
INR	1.4 (1.2–1.7)
Creatinine [mg/dl]	.8 (.7–1.2)
Bilirubin [mg/dl]	1.6 (.8–3.3)
Albumin [g/dl]	2.7 (2.3–3.2)
Sodium [mmol/l]	137 (133–140)
ALT [U/l]	41 (27–63)
AST [U/l]	74 (47–130)
FIPS	−.27 (−1.15–.51)
MELD	13 (10–19)
MELD‐sodium	16 (12–22)
MELD 3.0	16 (12–22)
Child–Pugh score	9 (8–10)
Child–Pugh stage	
A	126 (11.1)
B	596 (52.6)
C	411 (36.3)
CLIF‐C AD score	52 (46–59)
Risk category CLIF‐C AD	
≤45	268 (23.7)
46–59	587 (51.8)
≥60	278 (24.5)
Mortality	
90‐day	201 (17.7)
180‐day	276 (24.4)
1‐year	349 (30.8)
Liver transplantation	23 (2.0)
TIPS implantation	84 (7.4)
Indication TIPS	
Refractory ascites	32 (38.1)
Secondary prophylaxis variceal bleeding	30 (35.7)
Other^b^	22 (26.2)

Abbreviations: ALT, alanine‐aminotransferase; AST, aspartate‐aminotransferase; CLIF‐C AD score, Chronic Liver Failure‐Consortium Acute Decompensation score; FIPS, Freiburg Index of Post‐TIPS Survival; HBV/HCV, chronic hepatitis B/C virus infection; HE, hepatic encephalopathy; INR, international normalized ratio; MASLD, metabolic dysfunction associated steatotic liver disease; MELD, Model of End‐Stage Liver Disease; TIPS, transjugular intrahepatic portosystemic shunt; WBC, white blood cell count.

^a^ 98 patients had additional alcoholic liver disease.

^b^ Pre‐emptive TIPS, portal decompression before surgery or portal vein thrombosis.

^c^ Number of decompensation features at time of inclusion such as ascites, variceal haemorrhage, HE, SBP, or any combinations thereof.

One‐year mortality in the study cohort was 30.8%, 180‐day mortality was 24.4% and 90‐day mortality was 17.7%. The corresponding Kaplan–Meier curve is shown in Figure S1. Only a negligible number of patients underwent liver transplantation during the 1‐year observation period (2.0%). TIPS placement was performed in a small proportion of patients (7.4%).

### 3.2 The FIPS is an independent predictor of 1‐year mortality

Next, predictors of 1‐year mortality in the study cohort were identified. The components of the FIPS (age, bilirubin, creatinine and albumin) each emerged as independent predictors of 1‐year mortality from multivariable Cox regression analyses (Table 2). Furthermore, INR and white blood cell count as well as ascites at inclusion and viral liver disease independently affected 1‐year mortality. Entering the FIPS into the Cox regression models confirmed that a higher FIPS was an independent predictor of 1‐year mortality (HR 1.806, 95% CI 1.632–1.998, *p* < .0001).

**TABLE 2:** Cox regression analyses of predictors of 1‐year mortality.

Parameters	Univariable regression			Multivariable regression		
	HR	95% CI	*p*‐value	HR	95% CI	*p*‐value
Age [years]	1.019	1.010–1.027	**<.0001**	1.039	1.029–1.049	**<.0001**
Male gender	1.138	.899–1.440	.2826			
Alcoholic liver disease	.936	.759–1.155	.5366			
Viral liver disease	1.139	.909–1.426	.2589	1.637	1.287–2.082	**<.0001**
Variceal bleeding^a^	.824	.652–1.039	.1021			
Ascites^a^	2.021	1.538–2.655	**<.0001**	1.701	1.291–2.241	.**0002**
SBP^a^	1.322	.984–1.775	.0636			
HE^a^	1.287	1.031–1.607	.**0259**			
Two or more signs of decompensation^a^	1.625	1.316–2.006	**<.0001**			
WBC [10^3^/μL]	1.062	1.045–1.078	**<.0001**	1.049	1.030–1.068	**<.0001**
INR	1.746	1.550–1.966	**<.0001**	1.590	1.365–1.852	**<.0001**
Bilirubin [mg/dL]	1.062	1.050–1.075	**<.0001**	1.052	1.037–1.068	**<.0001**
Albumin [g/dL]	.540	.456–.641	**<.0001**	.607	.507–.727	**<.0001**
Creatinine [mg/dL]	1.477	1.375–1.588	**<.0001**	1.295	1.191–1.408	**<.0001**
Sodium [mmol/L]	.980	.973–.988	**<.0001**			
FIPS^b^	1.978	1.797–2.178	**<.0001**	1.806	1.632–1.998	**<.0001**

Abbreviations: HE, hepatic encephalopathy; INR, international normalized ratio; SBP, spontaneous bacterial peritonitis; WBC, white blood cell count.

^a^ At baseline.

^b^ To avoid bias by variable interference, either the FIPS *or* its components age, bilirubin, creatinine, and albumin were entered into multivariable regression.

### 3.3 Discriminatory performance of the FIPS

**FIGURE 2:**
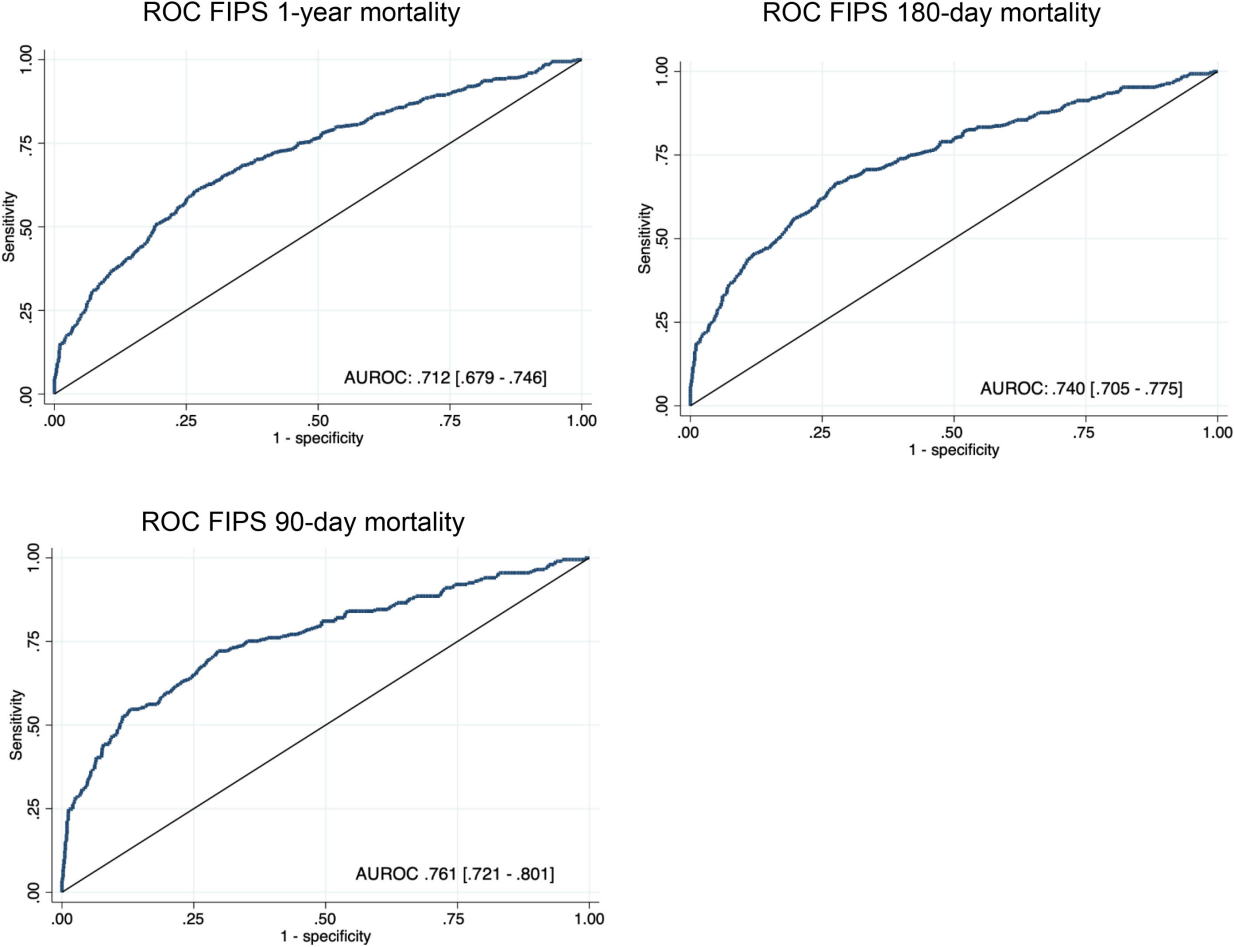
ROC analyses of the FIPS. The FIPS showed sustained discriminatory performance in the prediction of mortality at 1‐year, 180 days and 90 days after baseline. FIPS, Freiburg Index of Post‐TIPS Survival.

ROC analyses demonstrated that the FIPS allowed precise prediction of mortality in the study cohort, as the AUROC of the FIPS was .712 [.679–.746] for 1‐year mortality, .740 [.705–.775] for 180‐day mortality and .761 [.721–.801] for 90‐day mortality (Figure 2). In comparison with the other prognostic scores investigated, the FIPS' discriminatory performance was significantly better than that of the Child‐Pugh score and the MELD, but comparable to the performance of MELD‐sodium, MELD 3.0 and CLIF‐C AD score, as summarized in Table 3. Analysis of distinct subgroups of patients with AD revealed that the FIPS' performance was especially good in patients admitted with variceal bleeding (*n* = 364, 32.1%). In these patients, the FIPS allowed significantly improved prediction of 1‐year mortality and 180‐day mortality (AUROC: .782 [.724–.839] and .787 [.726–.849]) in comparison with Child–Pugh score (.717 [.657–.777], *p* = .0019 and .720 [.657–.782], *p* = .0118), MELD (.731 [.670–.792], *p* = .0131 and .743 [.678–.808], *p* = .0328), MELD‐sodium (.725 [.662–.789], *p* = .0074 and .738 [.672–.805], *p* = .0222), MELD 3.0 (.726 [.662–.790], *p* = .0042 and .738 [.670–.805], *p* = .0144) and CLIF‐C AD score (.724 [.660–.788], *p* = .0071 and .746 [.681–.812], *p* = .0496). In patients with leading ascitic decompensation at baseline (*n* = 637, 56.2%), however, the CLIF‐C AD score performed significantly better in the prediction of 90‐day mortality compared with the FIPS (AUROC .775 [.729–.822] vs. [.724 .671–.778], *p* = .0337). No such effect was observed in distinct analyses of patients with viral and alcohol‐related liver disease.

**TABLE 3:** Discriminatory performance of the FIPS versus other prognostic scores.

	AUROC [95% CI] 1‐year mortality	*p‐value*	AUROC [95% CI] 180‐day mortality	*p‐value*	AUROC [95% CI] 90‐day mortality	*p‐value*
All patients (*n* = 1133)						
FIPS	.712 [.679–.746]	Ref.	.740 [.705–.775]	Ref.	.761 [.721–.801]	Ref.
Child–Pugh	.669 [.635–.702]	**.0253**	.678 [.643–.714]	**.0020**	.718 [.679–.756]	**.0499**
MELD	.677 [.642–.712]	.**0150**	.708 [.671–.744]	**.0296**	.752 [.713–.792]	.5989
MELD‐sodium	.686 [.652–.721]	.0984	.715 [.678–.752]	.1238	.763 [.724–.802]	.8857
MELD 3.0	.686 [.651–.720]	.0831	.716 [.679–.753]	.1178	.765 [.726–.803]	.8118
CLIF‐C AD	.694 [.660–.727]	.2228	.733 [.698–.769]	.6670	.789 [.753–.826]	.0892
Patients with variceal bleeding at baseline (*n* = 364)^a^						
FIPS	.782 [.724–.839]	Ref.	.787 [.726–.849]	Ref.	.839 [.778–.900]	Ref.
Child–Pugh	.717 [.657–.777]	**.0119**	.720 [.657–.782]	**.0118**	.794 [.735–.853]	.1605
MELD	.731 [.670–.792]	**.0131**	.743 [.678–.808]	**.0328**	.800 [.734–.867]	.0878
MELD‐sodium	.725 [.662–.789]	**.0074**	.738 [.672–.805]	**.0222**	.795 [.727–.864]	.0514
MELD 3.0	.726 [.662–.790]	**.0042**	.738 [.670–.805]	**.0114**	.805 [.738–.873]	.1602
CLIF‐C AD	.724 [.660–.788]	**.0071**	.746 [.681–.812]	.0496	.826 [.766–.886]	.4732
Patients with ascites at baseline (*n* = 637)^a^						
FIPS	.683 [.639–.727]	Ref.	.721 [.675–.767]	Ref.	.724 [.671–.778]	Ref.
Child–Pugh	.621 [.576–.666]	**.0296**	.646 [.598–.694]	**.0118**	.667 [.613–.721]	.0871
MELD	.641 [.595–.687]	**.0455**	.688 [.640–.735]	.1293	.725 [.673–.777]	.9726
MELD‐sodium	.649 [.603–.695]	.1410	.698 [.650–.745]	.3255	.737 [.687–.788]	.6111
MELD 3.0	.647 [.602–.693]	.1166	.696 [.648–.743]	.2699	.731 [.681–.782]	.7781
CLIF‐C AD	.683 [.640–.726]	.9829	.730 [.686–.775]	.6826	.775 [.729–.822]	**.0337**
Patients with viral liver disease (*n* = 334)						
FIPS	.700 [.641–.760]	Ref.	.731 [.667–.794]	Ref.	.761 [.689–.833]	Ref.
Child–Pugh	.655 [.594–.716]	.1657	.656 [.589–.723]	**.0317**	.711 [.642–.780]	.1986
MELD	.671 [.609–.733]	.2076	.710 [.644–.775]	.3842	.770 [.702–.838]	.7236
MELD‐sodium	.670 [.608–.733]	.2387	.708 [.641–.774]	.3973	.774 [.706–.841]	.6487
MELD 3.0	.669 [.606–.731]	.1868	.710 [.643–.777]	.4054	.775 [.707–.843]	.5818
CLIF‐C AD	.675 [.614–.737]	.3851	.729 [.663–.794]	.9483	.808 [.741–.875]	.1219
Patients with alcohol‐related liver disease (*n* = 605)						
FIPS	.691 [.643–.738]	Ref.	.734 [.684–.784]	Ref.	.737 [.679–.796]	Ref.
Child–Pugh	.650 [.603–.696]	.1516	.656 [.606–.706]	**.0085**	.695 [.641–.748]	.1946
MELD	.672 [.624–.720]	.3674	.709 [.659–.760]	.2415	.734 [.677–.791]	.8840
MELD‐sodium	.680 [.632–.728]	.6447	.707 [.654–.759]	.2577	.740 [.683–.797]	.9327
MELD 3.0	.687 [.640–.734]	.8714	.714 [.663–.765]	.3991	.747 [.692–.802]	.7133
CLIF‐C AD	.686 [.639–.733]	.8252	.730 [.682–.779]	.8710	.775 [.724–.827]	.1308

Abbreviations: AUROC, area under the receiver operator characteristic; CI, confidence interval; CLIF‐C AD, Chronic Liver Failure Consortium Acute Decompensation (score); FIPS, Freiburg Index of Post‐TIPS Survival; MELD, Model for End‐Stage Liver Disease.

^a^ In patients presenting with both ascites and variceal bleeding at baseline, bleeding was considered as leading decompensating event.

Bold indicates significance level at *p* value < .05

### 3.4 Evaluation of a FIPS cut‐off value for risk stratification of patients with AD

**FIGURE 3:**
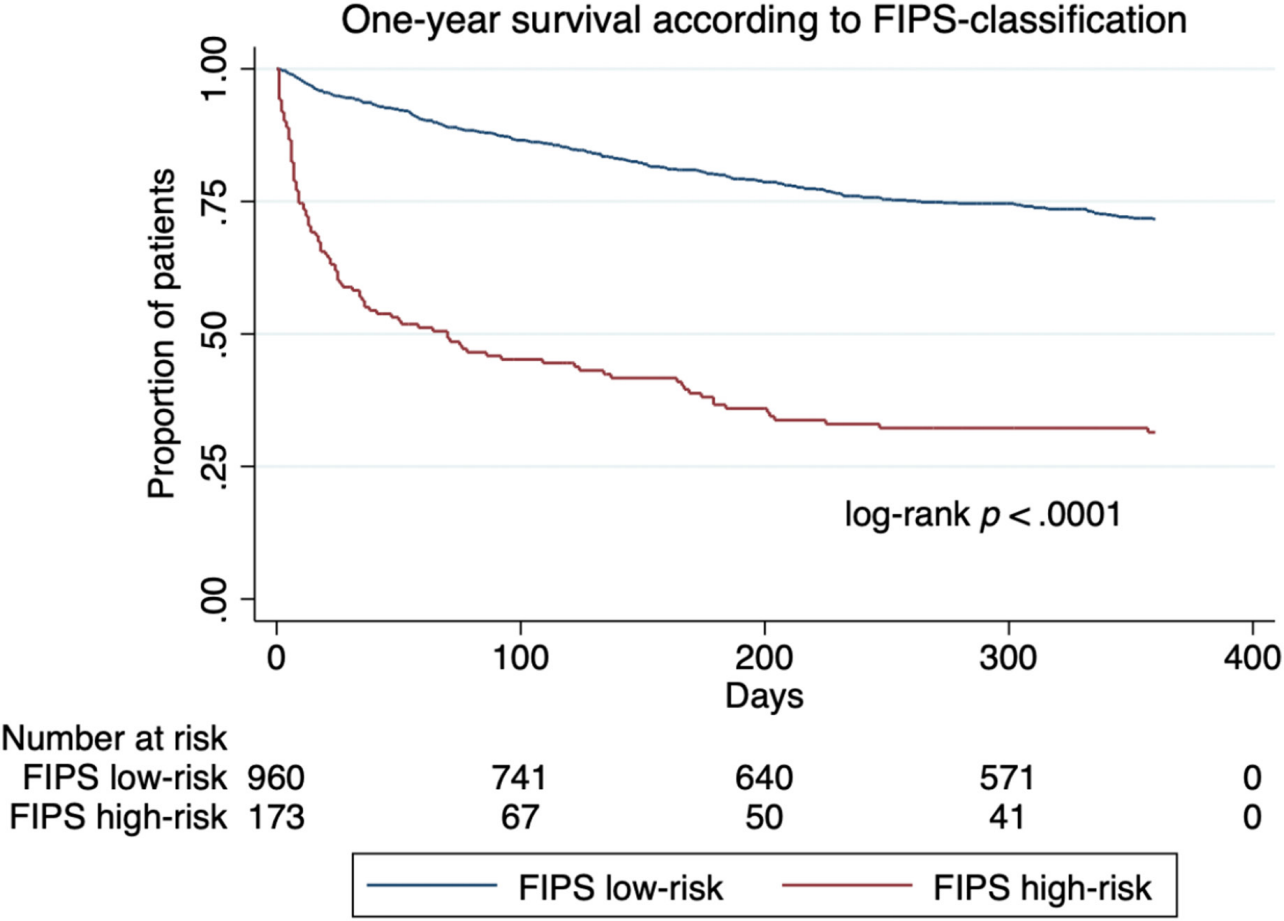
One‐year survival according to FIPS risk‐classification. Applying a FIPS cut‐off value of ≥.92 classified 173 patients (15.3%) as high‐risk and 960 patients (84.7%) as low‐risk group. One‐year survival was significantly lower in the high‐risk group compared to the low‐risk group. FIPS, Freiburg Index of Post‐TIPS Survival.

Next, a FIPS cut‐off value to stratify patients with AD according to mortality within 1 year was evaluated. In fact, with .95 the 85th percentile of the FIPS in the study cohort was almost identical to the FIPS cut‐off value of .92 determined in TIPS patients in the original publication of the FIPS (with only two patients having a FIPS between .92 and .95). Hence, for the sake of practicality, the cut‐off value of ≥.92 to define a high‐risk group was adopted for stratification of patients with AD. Application of this cut‐off value classified 173 patients (15.3%) as high‐risk and 960 patients (84.7%) as low‐risk group. In the high‐risk group, 1‐year mortality was 2.5‐fold higher (63.0% vs. 25.0%), 180‐day mortality was more than threefold higher (59.0% vs. 18.1%) and 90‐day mortality was more than fourfold higher (51.4% vs. 11.7%) in comparison with the low‐risk group (Figure 3).

## 4 DISCUSSION

The FIPS is a novel prognostic score that was developed to predict mortality in cirrhosis patients allocated to TIPS implantation for the treatment of refractory ascites or for secondary prophylaxis of variceal haemorrhage.^9^ Of note, these patients represent a specific subset among patients with advanced chronic liver disease, characterized by severe portal hypertension and a history of multiple episodes of complications related thereto. So far, the FIPS' prognostic relevance in other clinical settings of decompensated cirrhosis remains unclear. To address this important aspect, the present study investigated a large, multi‐centric cohort of 1133 patients with AD (not primarily allocated to TIPS implantation). Our analysis demonstrated that the FIPS accurately discriminated patients admitted with AD according to mortality within 1 year. Thereby, the FIPS' solid performance in the prediction of death was sustained over time, which was highlighted by an AUROC of ≥.700 for the prediction of 1‐year, 180‐day and 90‐day mortality. In our cohort of patients with AD, the FIPS allowed significantly better discrimination according to mortality compared with Child–Pugh score and MELD and equally good discrimination compared with MELD‐sodium, MELD 3.0 and CLIF‐C AD score. It is an important finding, however, that the investigated scores ranked differently in distinct subgroups of patients with AD. In fact, the FIPS proved to be superior to the other investigated scores in predicting long‐term mortality (at 1‐year and 180‐days) in patients presenting with variceal bleeding. Unfortunately, this study cannot provide a detailed analysis of the clinical course after the initial episode of AD or the cause of death for the included patients. It remains unclear why the FIPS is particularly suitable for stratifying patients with variceal bleeding based on their risk of death within 1 year. One plausible explanation is that the FIPS identifies patients who are at high risk of experiencing late re‐bleeding. This hypothesis should be further elucidated in future studies, as it could potentially impact the allocation of patients with variceal bleeding to TIPS implantation. In contrast to patients with variceal haemorrhage at baseline, the FIPS did not discriminate significantly better in patients with leading ascitic decompensation at baseline but was outperformed in the prediction of short‐term mortality (at 90 days) by the CLIF‐C AD score. These results indicate that refined strategies for different clinical phenotypes of patients with AD are necessary. Further, it is important to realize that with AUROCs between .669 and .712 all investigated prognostic scores involved a relevant number of erroneous predictions of mortality within 1 year after AD. This demonstrates that prognostic assessment of patients with AD remains a challenging issue. Further head‐to‐head investigation of prognostic scores in defined subgroups of patients with AD is necessary to achieve optimum risk‐stratification of these critically ill patients in clinical practice. The present study shows that the FIPS is a promising tool in this context that should be further evaluated.

Strengths of the present study are the large sample size and its multi‐centric design. An important limitation of this study is its retrospective design. Naturally, this holds the possibility of bias, as assessment of study data did not take place at defined study visits, but data including survival were retrospectively extracted from the medical records and with the help of civil registries. The fact that mortality rates in our patient cohort are comparable to those previously reported in patients with AD (for example in the PREDICT study cohort [*n* = 1071]: 90‐day mortality 16%, 1‐year mortality 27%^15^) supports the validity of our data. In any case, the present results should be validated in other cohorts of patients with AD in future studies.

In summary, the present study demonstrates that the FIPS is not only suitable for predicting mortality in patients with decompensated cirrhosis allocated to TIPS implantation, but also offers precise prediction of mortality within 1 year in patients with acutely decompensated cirrhosis. Hence, the FIPS is a comprehensive and objective approach for prognostic assessment of patients with decompensated cirrhosis. Our results propose that in clinical practice, the FIPS is especially useful to predict long‐term mortality in patients presenting with variceal bleeding. Follow‐up studies are needed to validate the findings of the present study and to further evaluate the performance of the FIPS in comparison with other prognostic scores.

## AUTHOR CONTRIBUTIONS

EK and LS were involved in study concept and design, analyses and interpretation of data. EK, LS, CG, OM and RK were involved in acquisition of data. EK, LS, DB and GA were involved in drafting of the manuscript. MS, JG, RK, LM, JH, MR, RT and SR were involved in critical revision of the manuscript for important intellectual content. DB and GA were involved in supervision.

## FUNDING INFORMATION

The authors received no financial support for this work.

## CONFLICT OF INTEREST STATEMENT

DB: Speakers' bureau of Gore and Falk Foundation; Travel grant: Gilead GA: Previous speaker Gilead, Abbvie and Gore, Grant: BMS.

## PATIENT CONSENT STATEMENT

All authors approved the final version of the article, including the authorship.

## Supporting information


Figure S1:

## Data Availability

The data presented in this study are available upon request from the corresponding authors.
