# Supplementary figures and images for: The Freiburg Index of Post‐TIPS Survival accurately predicts mortality in patients with acute decompensation of cirrhosis
Source: Liver Int. 2024 Sep 9;44(12):3229–37. doi: 10.1111/liv.16098 (PMC11586886; doi:10.1111/liv.16098)

## Slide 1
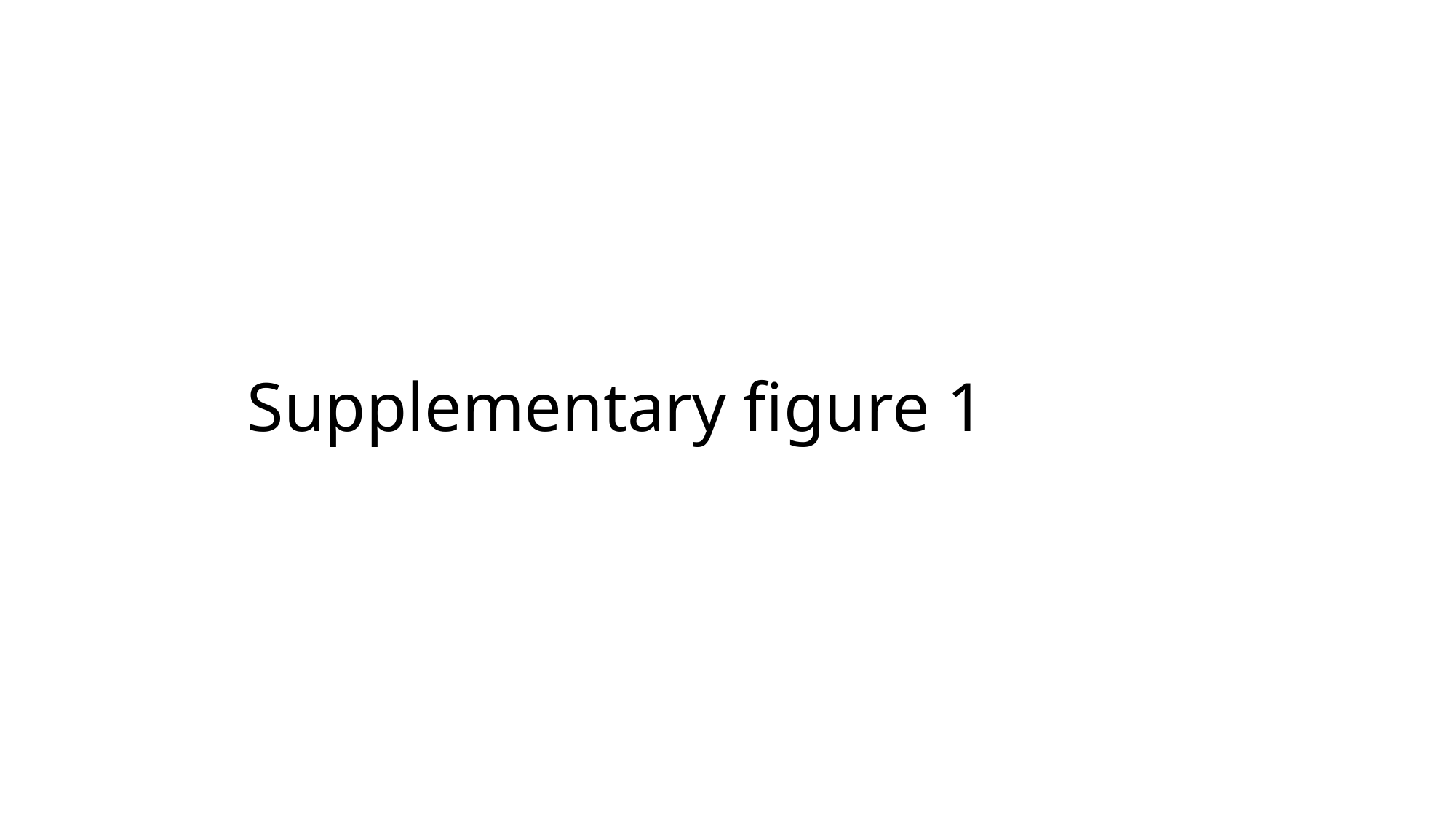

# Supplementary figure 1

## Slide 2
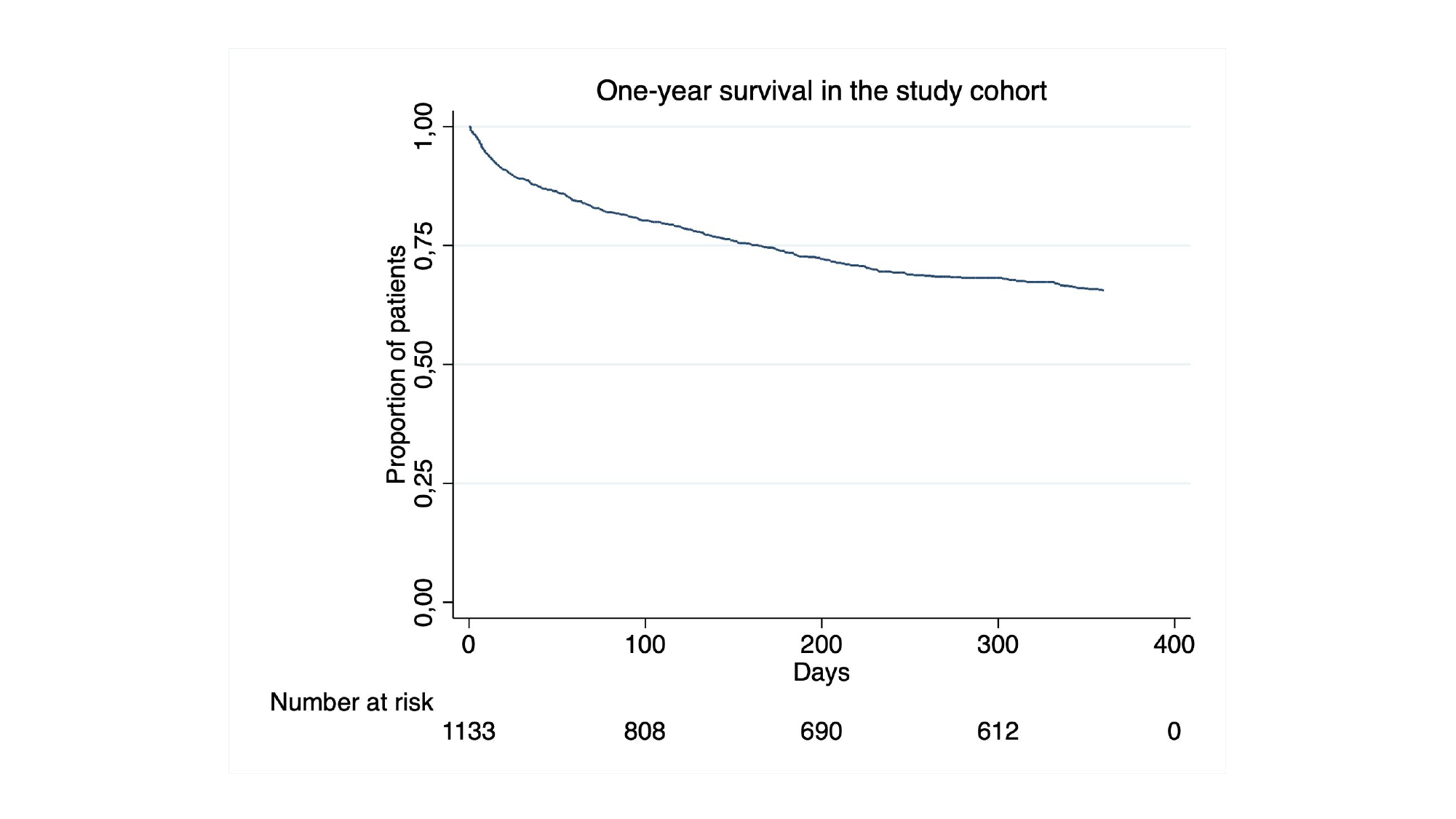

Supplement: Supplementary file 1 — Figure S1: [file LIV-44-3229-s001.pptx]
